# Statistical Shape Analysis of Ascending Thoracic Aortic Aneurysm: Correlation between Shape and Biomechanical Descriptors

**DOI:** 10.3390/jpm10020028

**Published:** 2020-04-22

**Authors:** Federica Cosentino, Giuseppe M Raffa, Giovanni Gentile, Valentina Agnese, Diego Bellavia, Michele Pilato, Salvatore Pasta

**Affiliations:** 1 Promozione della Salute, Materno-Infantile, di Medicina Interna e Specialistica di Eccellenza “G. D’Alessandro”, Piazza delle Cliniche, University of Palermo, n.2, 90128 Palermo, Italy; 2 Department for the Treatment and Study of Cardiothoracic Diseases and Cardiothoracic Transplantation, IRCCS-ISMETT, via Tricomi n.5, 90127 Palermo, Italy; vagnese@ismett.edu (V.A.); bellavia@ismett.edu (D.B.); pilato@ismett.edu (M.P.); 3 Department of Diagnostic and Therapeutic Services, Radiology Unit, IRCCS-ISMETT, via Tricomi n.5, 90127 Palermo, Italy; gentile@ismett.edu; 4 Department of Engineering, Viale delle Scienze, Ed.8, University of Palermo, 90128 Palermo, Italy; salvatore.pasta@unipa.it

**Keywords:** shape analysis, principal component analysis, shear stress: strain, computational modeling

## Abstract

An ascending thoracic aortic aneurysm (ATAA) is a heterogeneous disease showing different patterns of aortic dilatation and valve morphologies, each with distinct clinical course. This study aimed to explore the aortic morphology and the associations between shape and function in a population of ATAA, while further assessing novel risk models of aortic surgery not based on aortic size. Shape variability of *n* = 106 patients with ATAA and different valve morphologies (i.e., bicuspid versus tricuspid aortic valve) was estimated by statistical shape analysis (SSA) to compute a mean aortic shape and its deformation. Once the computational atlas was built, principal component analysis (PCA) allowed to reduce the complex ATAA anatomy to a few shape modes, which were correlated to shear stress and aortic strain, as determined by computational analysis. Findings demonstrated that shape modes are associated to specific morphological features of aneurysmal aorta as the vessel tortuosity and local bulging of the ATAA. A predictive model, built with principal shape modes of the ATAA wall, achieved better performance in stratifying surgically operated ATAAs versus monitored ATAAs, with respect to a baseline model using the maximum aortic diameter. Using current imaging resources, this study demonstrated the potential of SSA to investigate the association between shape and function in ATAAs, with the goal of developing a personalized approach for the treatment of the severity of aneurysmal aorta.

## 1. Introduction

Diagnosis and risk stratification of ascending thoracic aortic aneurysms (ATAA) are primarily based on medical imaging analysis, predisposing factors and patient familiarities [1]. The assessment of the maximum aortic diameter of an ATAA by imaging is necessary to understand whether elective repair is needed to avoid fatal complications, such as rupture or dissection. If left untreated, the risk of fatal complications can be as high as 50% in patients with a large ATAA wall (aortic diameter > 50 mm) [2,3]. Although ATAA is a relatively rare disease with an estimated incidence of 5.0 per 100,000 individuals per year [4], a dilated aorta is a common finding in patients with the congenital bicuspid aortic valve (BAV), as opposed to patients with the morphologically normal tricuspid aortic valve (TAV) [5]. Clinical evidence has shown that BAV ATAA is a markedly heterogeneous entity, with aortic dilatation occurring in the aortic root, the tubular ascending aorta, the proximal aortic arch or any combination of these types of dilatations [6,7]. These patterns of aortic dilatations are also associated to different BAV phenotype (i.e., the anterior-posterior cusp fusion or the right-left cusp fusion), each leading to ATAAs with distinct clinical outcome. The awareness of the heterogeneous nature of the aortopathy has led to the development of new classification schemes to interpret different BAV morphotypes and patterns of aortic dilatation, and to ultimately stratify the severity of ATAAs [8,9]. None of the proposed classification schemes has shown good prognostic significance, because they have failed to capture the full three-dimensional (3D) shape of an ATAA. Besides phenotypic criteria, novel principles for risk stratification are emerging to overcome the paradox of the current clinical criterion, based on the maximum aortic diameter of the ATAA. Patients undergo elective repair of the ATAA on the basis of the size and progression rate of the aortic diameter, but aortic size has a limited predictive value, as adverse events may occur when aortic diameters are <55 mm, and even <45 mm [10]. Among novel approaches for risk stratification, computational modeling has shown promise in the estimation of ATAA by means of prediction of intramural stress [11] and shear stress [12,13,14]. 

It is therefore necessary to explore the ATAA shape variability and go beyond risk assessment merely based on cross-sectional measurements of the dilated aorta. The abundance of 3D information provided by medical imaging can be fully exploited using a modern statistical shape analysis (SSA) methodology to quantitatively estimate the morphology of the aneurysmal ascending aorta. This approach makes it possible to visualize and quantify the variability of the aneurysm shape, including global and local geometrical patterns in a patient population of ATAAs [15,16,17]. The shape variability can be represented by a computational atlas, describing all anatomical shape information and its variations around a mean shape or template. Once the computational atlas is built, predictive statistical models can be developed to explore how changes in shape are associated to clinically-measurable anatomic characteristics (i.e., ATAA diameter) or functional parameters of the aneurysmal aorta. 

This study aimed to explore nuances in 3D aortic morphology in a cohort of patients with ATAA and assess correlation with aneurysm function. The principal modes of aortic shape were extrapolated by principal component analysis (PCA) to retain the most important shape modes that are responsible for the ATAA-related geometrical variability. Then, the correlation of shape modes with clinically-measureable anatomic variables and biomechanical descriptors was explored. Assuming that wall shear stress (WSS) and aortic wall strain have an important implication for the development and progression of the ATAA, computational analyses were performed to estimate how such important biomechanical parameters can vary with changes in the aortic dilatation. Logistic regression was adopted to develop a predictive model of the risk of ATAA surgery based on main shape modes. This study proves the potential of SSA to assess the association between shape and function, as well as to develop new risk models considering the complex anatomy of ATAAs.

## 2. Material and Methods

### 2.1. Study Population

After internal review board approval and informed consent, the ATAA shape variability of a total of *n* = 106 patients referred for aortic size measurement was evaluated by electrocardiogram-gated computed tomography angiography (CTA). Patients were stratified in two groups, according to the aortic valve morphology. Specifically, *n* = 53 patients had an ATAA with TAV, while *n* = 53 ATAAs had BAV with different phenotypes (i.e., anterior-posterior and right-left leaflet cusp fusion). As a control group, *n* = 19 individuals with non-aneurysmal aorta were included from organ donor and heart transplant recipients. None of the patients had both severe aortic stenosis and regurgitation, left ventricular dilatation, or evidence of uncontrolled stage II hypertension, as these were considered confounding variables of the decision to carry out surgery, as previously found by our group [18]. The primary end point was surgical repair of aorta and/or valve for maximum aortic diameter, although the timing for surgery was also influenced by other determinants, such as clinical predisposing factors, comorbidities, and family history. Table 1 summarizes clinical and demographic data of the patient study group.

### 2.2. Segmentation and Anatomical Measurements 

CTA images at both end-diastole and peak systole were segmented using semi-automatic thresholding and region growing techniques, combined with manual editing of masks in commercial software (Mimics v20, Materialize NV, Leuven, Belgium) as described previously [19,20,21,22]. The 3D ATAA models were cut near the brachiocephalic artery, to reduce irrelevant shape variability related to the aortic arch and descending aorta. Segmented models of ATAAs were stored as computational surface meshes for the SSA.

**Figure 1:**
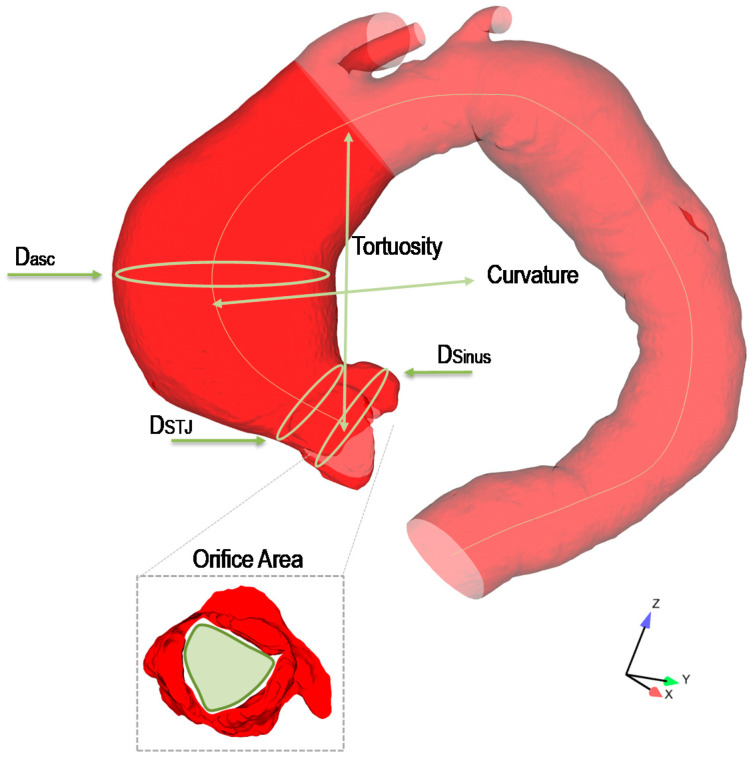
Sketch of an ascending thoracic aortic aneurysm (ATAA) showing measurements of aortic morphology taken for each patient.

For all patients, aortic diameters were measured at the Valsalsa sinuses, sino-tubular junction, and mid-ascending aorta, using standard imaging techniques (Figure 1). Aortic size was also used to group ATAAs in aortic dilatation at the aortic root (i.e., Type N), aortic dilatation of tubular portion of ascending aorta (i.e., Type A), and tubular involvement of ascending aorta (i.e., Type N), according to Schaefer’s classification scheme [23]. The aortic valve morphology was determined from CTA scans, reconstructed to obtain images at both diastole and systole. BAV ATAA were grouped according to the valve raphe in antero-posterior (AP) and right-left (RL) bicuspid patients. Ascending aortic curvature and tortuosity, orifice area at peak systole, and transaortic flow jet from echocardiography were also evaluated for each patient. Table 2 summarizes morphological features of the patient population.

### 2.3. SSA Method

SSA was performed using a custom algorithm developed in the mathematical language program MATLAB (R2018, MathWorks Inc., Natick, MA, USA). The mean aortic shape of patient population and variations around this mean were computed after (i) pre-processing of segmented ATAA surface meshes; (ii) automatic alignment based on several rigid registrations and transformations; (iii) PCA, followed by logistic regression and receiver operating characteristic (ROC) curves. 

Prior to alignment, the ATAA mesh surfaces were evenly sampled at sufficient resolution to capture all the shape features available for the ascending aorta. The surface sampling process resulted in 15,000 Cartesian (*xi*, *yi*, *zi*) points for each ATAA, as obtained after a convergence analysis of the resulting PCA shape modes. Starting from the original ATAA mesh surface from the segmentation, random sampling was carried out from low (2000 points) to high (25,000 points) mesh resolution. For each resolution, the first shape mode was plotted against the mesh resolution to assess the convergence when the change of the shape mode was <5%. 

Each sampled point data was rigidly aligned, using translation and rotation to an initial reference shape, using the iterative closest point algorithm. The initial reference shape was determined as the closest shape to the mean aortic diameter of ATAA population. This can, however, lead to an initial template shape that is quite biased with respect to the initial reference shape. Thus, a new set of shape transformations were done from the initial template shape to each rigidly aligned shape. The rigid alignment was therefore repeated, using the mean mesh as the reference shape. To reduce bias, the previous steps of rigid alignment, followed by shape transformation and, again, rigid alignment, were repeated a number of times until the average mesh did not change. Finally, the aligned 3D ATAA surface models were used as input for the PCA. 

The PCA methodology was applied to reduce the complex ATAA shape to few components, using the build-in function implemented in MATLAB. Using orthogonal transformations, PCA project the data onto a linear space of maximum variation directions, known as “shape mode” or “mode”. Shape modes are specific aspects of the anatomical variation of ATAA, and help to understand the morphological features that cannot be described by the aortic diameter alone. After projection, the number of retained modes is usually well below the number of original variables, yet retains a high percentage of the overall variability in the original set. The first mode accounts for as much of the variability in the data as possible, and each succeeding mode in turn has the highest residual variance possible showing specific anatomical features of ATAA shape. The coordinate of the surface sampling points (*xi*, *yi*, *zi*) were concatenated into a shape vector and assembled into a matrix. The eigenvectors of the covariance matrix formed the principal component modes, and their corresponding eigenvalues indicate the proportion of the total variance explained by each mode. The contribution of each mode can be visualized deforming the template from low −2 standard deviation (SD) to high +2 SD values of each mode’s deformation vector. Shape vectors numerically represent the contribution that each shape mode has on each ATAA, and were used for statistical analyses, thereby supporting the identification of specific shape features. 

### 2.4. Strain and Flow Analysis

Strain analysis of ATAA wall mechanics at peak systole was done using an algorithm previously developed by our group [20,24,25]. For each ATAA, the aortic surface mesh segmented at diastole was projected normally onto the aortic surface mesh at systole, and then the displacement field was achieved as the Euclidean distance between closest points. Thus, the relative displacement of the aortic wall characterizes the diastolic-to-systolic displacement field, assuming the diastole as the baseline configuration. For each point of ATAA wall, the systolic strain distribution can be calculated as the ratio of the relative displacement to the baseline configuration (i.e., the aortic surface at diastole). 

To study the correlation of shear stress with the morphological features, computational flow analyses were developed, according to our previously developed approach [21,26,27,28,29,30,31,32,33,34]. Hemodynamics were studied at the peak systole, with the aortic valve at the fully opened configuration. For each patient, the ATAA surface derived by CTA segmentation was meshed with unstructured tetrahedral elements at spatial resolution of 0.3 × 0.3 × 0.3 mm. The blood was assumed as an incompressible laminar-flow fluid with non-Newtonian viscosity described by the Carreau model. To include patient-specific hemodynamics, the transaortic jet velocity evaluated by Doppler echocardiography was set as the inflow velocity condition at aortic valve. For each outlet, the global vascular resistance and arterial compliance were estimated from echocardiographic and brachial pressure measurements. Then, these parameters were used to compute the outflow boundary conditions of a three-element Windkessel model coupled to each outflow branch. The Navier–Stokes equations governing fluid motion were solved with an implicit algorithm in FLUENT v19 (ANSYS Inc., Canonsburg, PA, USA).

### 2.5. Statistical Analysis

Data are shown as mean ± SD or percentage (number), depending on the variable distribution. The Mann–Whitney test was used to compare variables among groups, while the *X*^2^ test was adopted to analyze frequencies. Pearson’s correlation was performed to identify linear relationships of shape modes with biomechanical descriptors. Two PCAs were carried out: (a) all ATAA patients, including both BAV ATAA and TAV ATAA (*n* = 106), to assess shape variability induced by the ascending aortic shape; (b) the group of BAV ATAA (*n* = 53) versus TAV ATAA (*n* = 53), to assess differences induced by the aortic valve morphology.

After PCA, a logistic regression model was used to identify which modes were most associated with differences between surgically-operated and monitored patients. The weight of the shape modes (retained upon 90% of total variance) were used as a predictor for the classification of the patient class. ROC curves were plotted to compute the area under the ROC curve as an index of the predictive value of the regression model. Cluster analysis to assess whether shape modes leads subgroups with specific shape variations was also performed. Statistical analyses were performed using SPSS software (IBM SPSS Statistics v.17, New York, NY, USA), with all probability values considered significant at 0.05 threshold. 

## 3. Results

**Figure 2:**
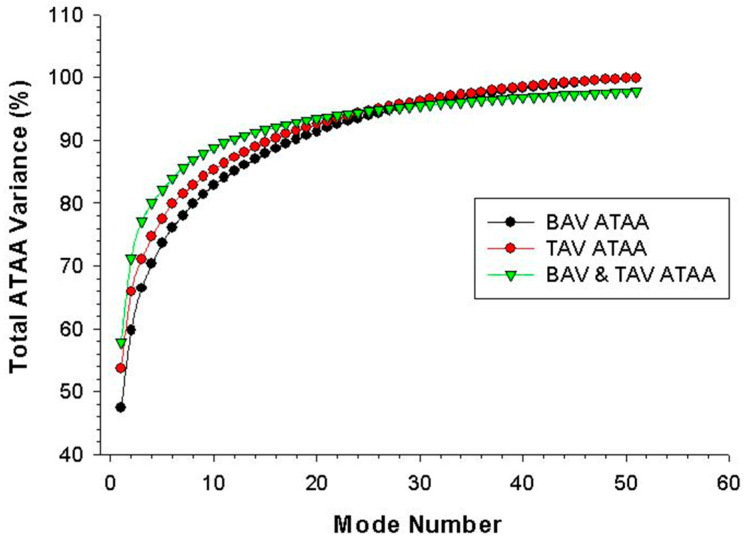
Scree plot of principal component analysis (PCA) analyses done for bicuspid aortic valve (BAV) & tricuspid aortic valve (TAV) ATAA together and for the separated groups of BAV ATAA and TAV ATAA.

**Figure 3:**
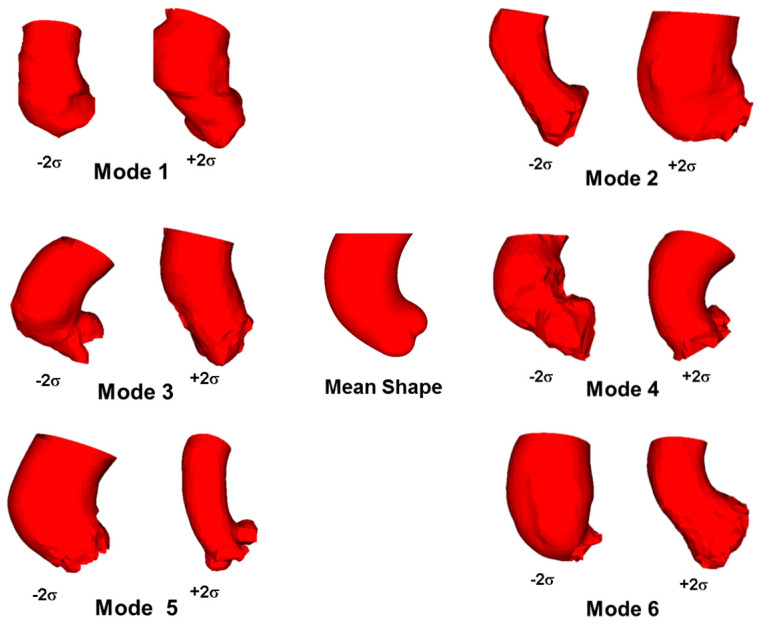
Dominant shape modes shown by deformations of the computed template from low (−2 SD) to high (+2 SD) values for BAV & TAV ATAA together.

Figure 2 shows the scree plot with cumulative variance, explained by each mode obtained for BAV & TAV ATAA together, and for separated groups of BAV ATAA and TAV ATAA. For all ATAA, the shape variation of aortic anatomy described by each mode is shown in Figure 3. The first six modes of shape variations represented 84% of the overall shape variability in the patient population, and hence the corresponding shape vectors were used for statistical analyses. Several shape modes were associated to different morphological features of ATAA, as that derived by visual assessment and correlation with morphological measurements. For the PCA with all ATAAs, we observed that the dominant shape feature of interest was Mode 1, which explained nearly 50% of the total variance, and was associated to the pattern of aortic dilatation (i.e., root phenotype, Type N, at −2 SD versus tubular ascending aortic dilatation, Type A, at +2 SD, see Figure 3).

**Figure 4:**
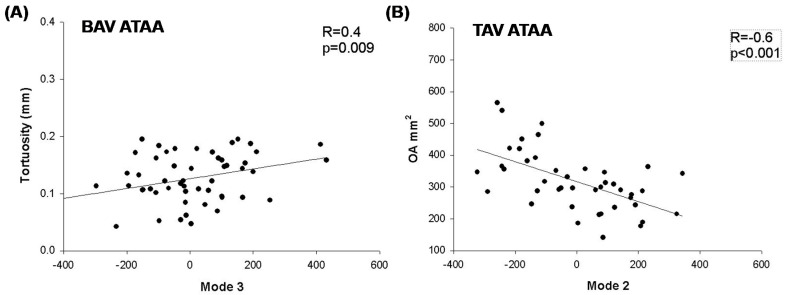
Correlations (**A**) between Mode 3 and vessel tortuosity for BAV ATAA and (**B**) between Mode 2 and orifice area for TAV ATAA.

When PCA was performed for groups stratified according to the aortic valve morphology, we observed that Mode 5 was associated to the overall size of BAV ATAAs. Mode 2 was negatively associated to the aortic valve orifice area of TAV ATAA (R = −0.6 and *p* < 0.001, Figure 4B), while Mode 3 was related to the vessel tortuosity of BAV ATAA (R = 0.4 and *p* = 0.009, see Figure 4A). Mode 4 was related to specifics characteristics of the dilated aorta, such as a bulged aortic dilatation.

**Figure 5:**
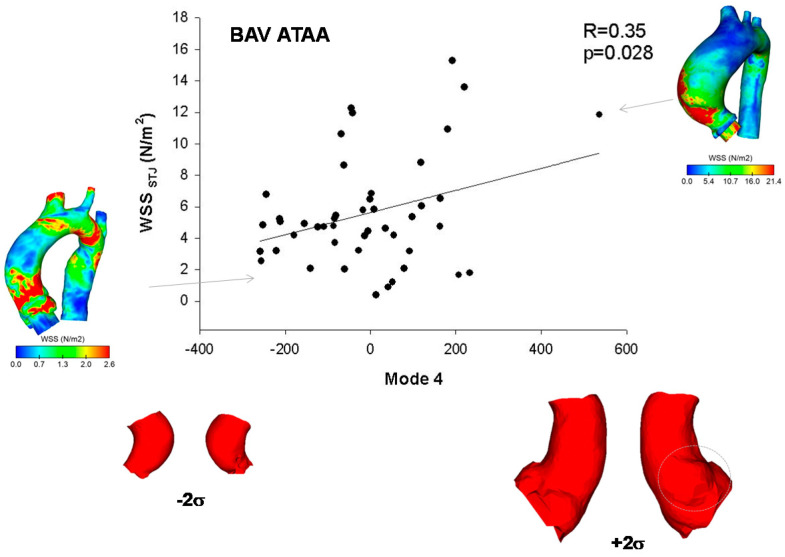
Correlation of wall shear stress (WSS) computed at STJ of BAV ATAA with Mode 4, as associated to a bulged dilatation of the anterolateral side of ATAA wall; map of WSS of patients being at extremity are also shown.

**Figure 6:**
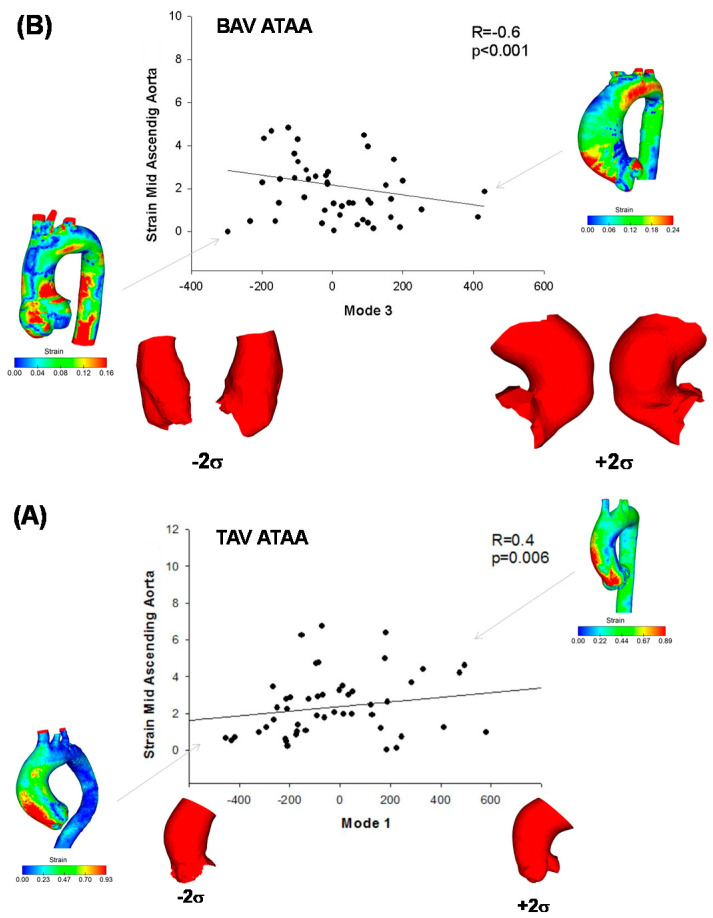
Correlation (**A**) between aortic strain computed at mid-ATAA of BAV patients with Mode 3, as associated to vessel tortuosity and (**B**) between aortic strain and mode 1 for TAV ATAA; map of aortic strain of patients being at extremity are also shown.

A positive correlation was found between mode 4 and WSS at STJ of BAV ATAA (R = 0.35, *p* = 0.028, Figure 5). While moving from low to higher WSS, the BAV ATAA is hence accompanied by larger aortic diameter with bulge dilatation in the anterior side of the ATAA wall just above STJ. Similarly, peak systolic strain at mid-ascending aorta of BAV ATAA was negatively correlated with changes in shape Mode 3 (R = −0.60, *p* < 0.001, Figure 6A) and thus to the vessel tortuosity. For TAV ATAA, Mode 1 had statistically significant correlation with peak systolic strain at mid-ascending aorta (R = 0.43, *p* = 0.006, Figure 6B). 

**Figure 7:**
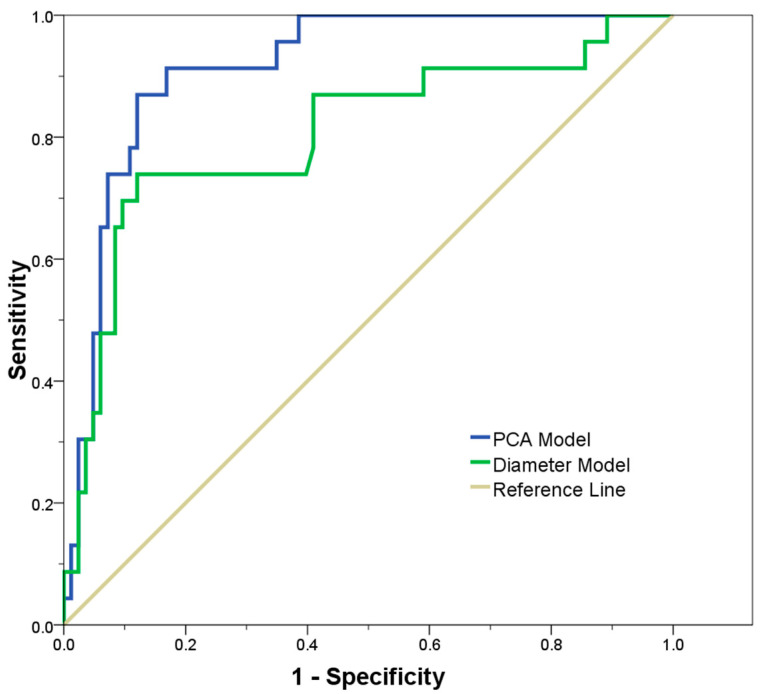
ROC curves for predicting the risk of surgery using a model based on principal shape modes as compared to the baseline model based on maximum aortic diameter.

**Figure 8:**
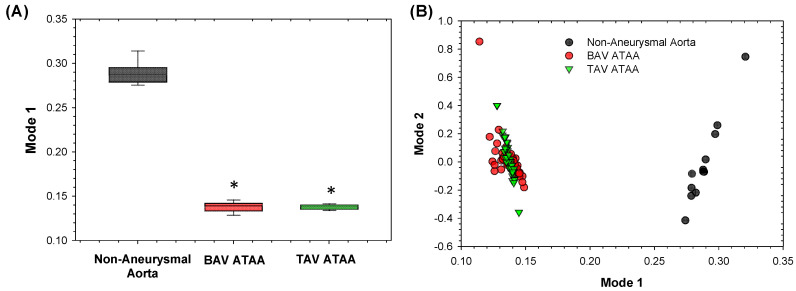
(**A**) box plots of mean values of Mode 1 for all groups; * denotes statistically significant difference with non-aneurysmal aorta; (**B**) revealed grouping for all groups.

A logistic regression model was studied to determine the probability of aortic surgery, on the basis of shape modes retained upon 90% of shape variability. This model was then compared to a baseline model, using the aortic diameter as predictor of surgery. ROC curves demonstrated that the principal shape modes of ATAA-related geometric variation can predict with high sensitivity and specificity the probability of aortic surgery (AUC = 0.914), as compared to the baseline model built only with the aortic diameter (AUC = 0.805, see Figure 7). Finally, the scatterplot of subject-specific shape Mode 1 versus Mode 2 highlighted a group stratification between non-aneurysmal aorta and ATAA, but no separation was observed for BAV ATAA versus TAV ATAA, as shown by Figure 8.

## 4. Discussion

In this study, we have presented an SSA of the ascending aneurysmal aorta built from a large dataset of CTA scans, including *n* = 106 patients with different valve morphologies. This framework allowed us to extract unique shape modes that visually and numerically characterize complex shape features that are otherwise impossible to capture using measurements of the aortic diameter. The extracted shape modes were related to biomechanical descriptors to shed light into shape and function, and to ultimately predict disease progression using a personalized approach, rather than a crude measurement of the patient’s aortic diameter. 

Currently, shape analysis was mainly performed for cardiac disorders to describe morphological descriptors of the left and right ventricular chambers [35,36,37]. Machine learning of ATAA shape and biomechanical parameters was also proposed [38,39,40]. From a clinical perspective, SSA does not require any manual process that could interfere with the variability assessment, and can be easily interpreted by radiologists. These mathematical approaches were based on shape-related parametrization by landmarks, as well as non-parametric frameworks showing a mean template and its deformation [41]. We demonstrated that shape modes are not only able to represent the overall changes of aneurysm phenotype and dimension (Mode 1 and 5), but also the vessel tortuosity (Mode 3) and local changes in the aortic wall geometry as a bulged aortic dilatation (Mode 4). The analysis of ATAA morphology exhibited a moderate dispersion that required upon six shape modes to achieve 84% of all geometric variability (and twelve modes for 90% of variability). This demonstrates that the ATAA is a quite heterogeneous disease and needs detailed 3D examination. Nearly 50% of total geometric variability in ATAAs can be attributed to a proportional size change (i.e., Mode 1), and this is in agreement with findings reported by Casciaro et al. [17] for the normal healthy aorta. They also documented that the second shape mode is related to aortic unfolding, as defined by an increased aortic arch tortuosity and height, and by a decreased aortic arch width. Aortic unfolding may determine the development of aortic dilatation and lengthening with ageing, accelerated by hypertension [42]. Ex vivo biomechanical testing has demonstrated no mechanical vulnerability of BAV ATAA, suggesting a conservative approach for the management of bicuspid aortopathy, such as that of TAV ATAA [43,44]. Using SSA, Sophocleous et al. [16] determined that morphological features of the aorta can be related to the hemodynamic impairment induced by aortic coarctation in patients with BAV. Later, Bruse et al. [15] highlighted that a high ejection fraction correlates with a more compacted, rounded aortic arch shape with a slim descending aorta, while a low ejection fraction is seen in patients with a more gothic arch shape and a slightly dilated descending aorta. These relationships between shape and function were assumed to be responsible for the adverse hemodynamic environment typically occurring in the descending aorta of patients with coarctation. 

Most interestingly, the association of Mode 3 and 4 with shear stress and strain in BAV and TAV ATAAs suggests that patients with bulged aortas have a high risk of developing high shear stress, while patients with a more tortuous ATAA have high aortic wall strain. It is recognized that increased tortuosity might be a marker for vascular fragility and a predictor of aortic dissection in patients with Marfan or BAV [45]; however, no relationship between strain and vessel tortuosity is known. The relationship here reported between shapes and biomechanical descriptors highlights that our methodology could be potentially used to detect outlying shapes in a complex and heterogeneous population, such as that of bicuspid aortopathy -which, in turn, might be associated to outlying functional behavior. Cardiac 4D flow MRI [46] and computational modelling [21,47] revealed the need for flow analysis of the aneurysmal aorta, with the potential to stratify patients at high risk of aortic complications. Shear stress was also found as an important regulator of extracellular matrix function by mechanotransduction [48,49]. Specific local changes of the ATAA wall, such as a bulged aorta, are commonly considered clinical evidence supporting the hemodynamic theory of aneurysm development, as opposed to the genetic theory. We speculate that the relationship between bulged ATAA shapes and high shear stress is the expression of the hemodynamic contribution of aneurysm development in our patient population, where the flow dictated by valve morphology locally impinges the anterolateral ATAA wall, and thus lead to the focal dilatation of ascending aorta. 

Clinical observations have also demonstrated varying degrees of patterns of aortic dilatation, each with distinct clinical course. The most common pattern of aortic dilatation involves the tubular portion of the ascending aorta, and is associated with an older age at diagnosis, while the aortic root phenotype occurs at a younger age, and is caused by a developmental defect [6]. This heterogeneity of ATAA disease renders the clinical decision-making process particularly challenging. For instance, the aortic root dilatation phenotype represents the more malignant and rapidly progressive disease that should be treated differently from the tubular aortic dilatation pattern. In this study, using logistic regression analysis and ROC curves, we demonstrated the value of a detailed 3D assessment of the complex shape of ATAAs to predict the likelihood of aortic surgery on a more personalized fashion. A predictive model built with principal shape modes of the ATAA wall achieved the best performance to predict the risk of surgery with respect to the risk-model based on the maximum aortic diameter. In this way, we have proven how it is possible to maximize the potential of existing imaging resources for outcome prediction, by using shape analysis at minimal human intervention. This approach could be also integrated with machine learning algorithms for the purpose of developing robust risk models and redefining the subgroup of patients at increased risk of ATAA complications. The search for robust risk models using novel shape-based biomarkers is particularly lively and of critical importance, given the paradox of the aortic diameter to fail a prognosis of aneurysm disease progression. 

The study has the disadvantages of a retrospective design in a heterogeneous disease, with limited number of surgically-operated patients. Gender differences and the presence of hypertension could have influenced the resulting shape modes. Additional shape analyses for subgroups of patients stratified for gender or the presence of hypertension should be performed to assess the impact of other clinical and demographics variables on the aortic shape features. However, the findings evinced the potential of the proposed SSA to investigate the association between ATAA shape and function. The study could be extended to the investigation of other biomechanical descriptors of ATAA. 

## 5. Conclusions

In this study, complex shape features of ATAAs were extracted by SSA and then correlated to clinical data and biomechanical descriptors to shed light on shape and function. Using a predictive model, we demonstrated the value of a detailed 3D assessment of the complex shape of the aneurysmal aorta to predict the risk of aortic surgery on a more personalized fashion.

## Figures and Tables

**Table 1:** Clinical and demographic characteristics of study population. Mann-Whitney test with α = 0.05.

Patients Characteristics	BAV ATAA	TAV ATAA	*p*-Value
*N*. subjects	53	53	
Age (years)	58 ± 1	65 ± 1	0.390
Male (%)	85.0	63.9	0.234
Surgery (%)	28	13	0.049
BSA (m^2^)	3.5 ± 6.2	2.4 ± 3.5	0.078
HR (bpm)	72.9 ± 10.8	72.8 ± 13.0	0.769
Psys (mmHg)	136.7 ± 12.5	135.3 ± 13.3	0.700
Pdias (mmHg)	77.3 ± 9.3	75.9 ± 9.6	0.964
MAP (mmHg)	93.4 ± 9.5	91.9 ± 8.1	0.107
SV (mL)	77.7 ± 30.8	77.1 ± 26.9	0.455
CO (L/min)	5.5 ± 2.2	5.5 ± 2.5	0.952
Hyper (%)	51.5	60.2	0.987
AI (%)			
None	7.1	9.2	0.721
Mild	15.1	34.0	0.082
Moderate	30.1	4.4	0.023
Severe	18.9	47.2	0.043
AS (%)			
None	21.1	0.0	1.000
Mild	7.8	0.0	1.000
Moderate	2.5	0.0	1.000

**Note**: BSA = body surface area; HR = heart rate; Psys = systolic blood pressure; Pdias = diastolic blood pressure; MAP = mean arterial pressure; SV = stroke volume; CO = cardiac output; Hyper = hypertension; AI = aortic insufficiency; AS = aortic stenosis.

**Table 2:** Morphological characteristics of patient population. Mann-Whitney test with α = 0.05.

*Size and Shape Parameters*	BAV ATAA	TAV ATAA	*p*-Value
***Aortic Diameters*** (mm)			
***Sinus***	41.4 ± 5.4	41.3 ± 5.5	0.999
***STJ***	36.4 ± 4.9	35.98 ± 4.4	0.656
***Mid-Ascending Aorta***	44.6 ± 5.5	44.3 ± 5.0	0.397
***Aortic Shape*** (%)			
***Type N***	32	38	0.887
***Type A***	57	58	0.999
***Type E***	11	4	0.034
***Aortic Curvature*** (L/mm)	0.03 ± 0.01	0.03 ± 0.01	0.743
***Aortic Tortuosity*** (/)	0.13 ± 0.04	0.12 ± 0.04	0.143
***BAV Aortopathy***			
***AP***	38	/	
***RL***	12	/	
***Orifice Area*** (mm^2^)	347.3 ± 88.5	318.6 ± 94.6	0.077
***Aortic Flow Jet*** (m/s)	1.9 ± 0.6	1.4 ± 0.3	0.006
